# Narcissus’ belief about his body: Aspects of narcissism, body image, and eating disorder symptoms

**DOI:** 10.1371/journal.pone.0293578

**Published:** 2023-11-09

**Authors:** Piotr Szymczak, Daniel Talbot, Emanuela S. Gritti, Peter K. Jonason

**Affiliations:** 1 Institute of Psychology, Cardinal Stefan Wyszyński University in Warsaw, Warsaw, Poland; 2 Department of Psychiatry, Westmead Hospital, Westmead, Australia; 3 University of Sydney, Camperdown, Australia; 4 University of Notre Dame, Fremantle, Australia; 5 Department of Psychology, University of Milano Bicocca, Milan, Italy; 6 Department of General Psychology, University of Padua, Padua, Italy; University of Valencia: Universitat de Valencia, SPAIN

## Abstract

**Objective:**

Narcissism may play a role in shaping body image concerns. Here we examined the relationships between narcissism (i.e., agentic extraversion, antagonism, narcissistic neuroticism, leadership/authority, exhibitionism/entitlement) and body image concerns and disturbances (i.e., drive for thinness, drive for muscularity, eating disorder symptoms, body mass index, current/desired fat, and current/desired muscularity).

**Methods:**

Mechanical Turk workers from the USA (*N* = 430; 64% male) completed the Narcissistic Admiration and Rivalry Questionnaire, the Hypersensitive Narcissism Scale, the Narcissistic Personality Inventory, the Drive for Muscularity Scale, the Drive for Thinness Scale, the Eating Disorder Examination Questionnaire–Short, and the Sex-Specific Somatomorphic Matrixes.

**Results:**

All narcissistic factors were associated with a greater drive for thinness (except for leadership/authority) and for muscularity, more eating disorder symptoms, a greater desired body fat (except for leadership/authority), and a greater current muscularity. Greater agentic extraversion and exhibitionism/entitlement were associated with lower levels of current body fat, and greater antagonism was associated with a greater desired muscularity.

**Discussion:**

Notably, individual differences in narcissism appeared to be important in understanding body image concerns, broadly speaking. We found that narcissism may be associated with body image concerns among both sexes differently, and especially that drive for thinness was more related to narcissism in men. Our results emphasize the importance of narcissism in formulating and treating body image-related disorders for both men and women. Ultimately, narcissistic features of personality may be risk factors for developing and perpetuating body image concerns, and therefore should be considered in assessment, formulation, diagnosis, and treatment of eating disorders.

## Introduction

The fundamental characteristic of narcissism seems to be antagonism. Moreover, we can identify two main types of narcissism: grandiose narcissism (i.e., agentic extraversion) and vulnerable narcissism (i.e., narcissistic neuroticism). These three characteristics (i.e., antagonism, agentic extraversion, and narcissistic neuroticism) make up the Trifurcated Model of Narcissism which is believed to cover all aspects of narcissism [1–3]. Agentic extraversion is associated with more subjective well-being, initial popularity, and attractiveness [4, 5], while narcissistic neuroticism may be linked to negative emotionality, interpersonal sensitivity, and psychoticism [5, 6]. Antagonism, as a core of the narcissistic personality trait, explains grandiose and vulnerable narcissism’s association with many interpersonal pathologies and stands for lack of empathy [5]. Apart from that, much attention has been devoted to such popular aspects of narcissism as leadership/authority and exhibitionism/entitlement [7], which can fit into Trifurcated Model of Narcissism as well whereby the leadership/authority portion is part of agentic extraversion, and the exhibitionism/entitlement portion is part of antagonism. These factors of narcissism, which are characterized by attention seeking, feelings of shame, or self-focus [5], may play a role in shaping one’s body image concerns.

Body image concerns are negative evaluations of one’s body, and, in more severe cases, may manifest as eating disorders [8]. Furthermore, body image concerns vary in focus, but generally refer to the size and shape of a specific feature of one’s body, or overall body size/shape/weight [9]. Two types of body image concerns, which can co-occur, are particularly relevant for their noteworthy presence in most industrialized countries: drive for thinness and drive for muscularity. Drive for thinness refers to the desire to maintain or obtain a slender, low body-fat figure [10]. Drive for muscularity refers the desire to have a lean and toned body, and is associated with depression, bulimic behaviors, muscle dysphoria, and psychological distress [11].

Although investigations on the matter are somewhat limited, body image concerns and narcissism are related [12]. For instance, women with eating disorders report greater pathological narcissism compared to women without eating disorders (Waller et al., 2007), and pathological narcissism predicts behavioral indicators of eating disorders such as body checking [13] and excessive exercise [14], which are associated with a drive for thinness [15] and muscularity [16]. It is likely that drive for thinness and muscularity, as well as their accompanying eating disorders, correspond to a profound need for internal (from oneself) and external (from other people) validation of the narcissistic individuals. Consequent behaviors such as over-exercising and excessive dieting are part of an appearance-correcting strategy of body image coping and self-regulatory processes [17]. Indeed, vulnerable narcissism is linked to body shame and body dissatisfaction in women [18, 19]. The findings regarding grandiose narcissism have been unequivocal, with modest evidence of a link with eating disorders suggesting potential implications for body image concerns, such as drive for muscularity, especially in men [20]. To our knowledge, only one study has investigated the contribution of more specific aspects of narcissistic personality—narcissistic admiration and rivalry [4]—to body image disturbances [19], finding moderate positive correlations between admiration and drive for thinness and drive for muscularity. Additionally, rivalry was negatively correlated with drive for thinness, and positively correlated with drive for muscularity. This study was limited in that they only studied these constructs in women and did not directly examine eating disorder symptoms (e.g., restriction, purging, and binge-eating).

Based on the above, it seems that individual differences in different aspects of narcissism might better inform us about one’s body image concerns and eating disorder symptoms than previous studies. Therefore, in our study we provided a broadband assessment of the relationships between different aspects of narcissism and body image. To best test this idea, we examined narcissism and body image in a multimethod-multimeasure matrix with several measures of narcissism and several measures of self-reported body image. Generally, consistent with sex-specific ideal bodies, men tend to report a greater drive for muscularity, whilst women tend to endorse a greater drive for thinness [21, 22]. However, notable exceptions to this pattern exist. The body with well-defined muscles, appearing toned and fit is being increasingly endorsed by women [23], while men report a desire to be leaner to improve the definition of their muscles [24]. Based on that, we test whether the associations differ in each sex and across the correlated measures of narcissism. Although, our study was, in part, exploratory in nature, we hypothesized that while narcissistic neuroticism, which is related to higher distress, may unprofitably influence one’s body image concerns and lead to eating disorders, agentic extraversion (together with its leadership/authority subfactor), on the other hand, which is related to power-seeking [2] may have the opposite effect, serving as a protective factor for body image disturbances and eating disorders. Antagonism, along with its exhibitionism/entitlement subfactor, constitutes a central aspect of narcissism and primarily drives the connections between narcissism and psychoticism [3]. Therefore, it should exhibit the most substantial links to concerns about body image and eating disorders.

## Method

### Participants and procedure

The sample was 430 adults (64% male) from the USA. Participants were recruited through Amazon Mechanical Turk (mTurk), an online platform often used for research due to its accessibility and diverse participant pool [25]. While mTurk has been subject to some controversy, it offers advantages in participant diversity and cost-efficiency. We implemented strict inclusion criteria (age: 18+, proficiency in English) and quality control measures, with an average survey completion time of 923.03 seconds (approximately 15 minutes) and a standard deviation of 686.43 seconds (approximately 11 minutes). Participants who didn’t complete all survey items, those who gave the same response for every item (e.g., all "1" on Likert scales), and those who provided unrealistic answers to open questions (e.g., age = "1234") were excluded to ensure data quality and mitigate potential issues associated with mTurk’s use [26]. This allowed us to harness the benefits of mTurk while maintaining data integrity. *A priori* power (*r* = .20; α = .05; 1-β = .80; 2-tailed distribution) analysis using G*Power software indicated that we had sufficient power to detect weak—albeit common in social and personality psychology—effects for our correlational (bivariate normal model; exact test family), comparisons for dependent correlations (two dependent Pearson’s *r*’s, common index; *z* test family), and comparisons for independent correlations (two independent Pearson’s *r*’s; *z* test family). Participants were aged between 18 and 72 years (*M* = 36.76, *SD* = 10.08), and predominantly (84%) heterosexual and self-identified as racially/ethnically “White” (73%). Participants reported their height in feet, inches, or centimeters, and their weight in pounds, ounces, or kilograms. If needed, height and weight were converted to centimeters and kilograms, respectively, and used to calculate body mass index (BMI; kg/m^2^). Participants received US$1.20 for their involvement in the study. Before the study, the tasks, and research procedure were explained to the participants, and all participants provided informed consent before proceeding to the study. Ethics approval for the study was granted by institutional Human Ethics Research Board at the University of Notre Dame, Sydney, Australia (ID: 2021-010S, approved February 2021), and the data is available on the Open Science Framework (https://osf.io/urpv7/).

### Measures

To measure individual differences in agentic extraversion and antagonism [3] we used the 18-item Narcissistic Admiration and Rivalry Questionnaire [4]. Participants reported their agreement (1 = *do not agree at all*; 6 = *completely agree*) with items like “I am great” (i.e., admiration) and “I want my rivals to fail” (i.e., rivalry). Items were averaged to create indexes of agentic extraversion (i.e., admiration) and antagonism (i.e., rivalry) with higher scores indicating greater endorsement of agentic extraversion, and antagonism.

To measure individual differences in narcissistic neuroticism [3, 27] we used the 10-item Hypersensitive Narcissism Scale [28]. Participants reported the extent to which items were characteristic of their feelings and behaviors (1 = *very uncharacteristic or untrue*; 5 = *very characteristic or true*) with items like “I often interpret the remarks of others in a personal way”. Items were averaged to create an index of narcissistic neuroticism with higher scores indicating greater endorsement of narcissistic neuroticism.

To measure individual differences in subfactors of agentic extraversion (i.e., leadership/authority) and antagonism (i.e., exhibitionism/entitlement [7, 29]) we used the 40-item Narcissistic Personality Inventory [30]. Respondents were asked to choose between narcissistic (e.g., “Modesty doesn’t become me”) and non-narcissistic (e.g., “I am essentially a modest person”) self-descriptions. Scores were summed to create indexes of leadership/authority and exhibitionism/entitlement with higher scores indicating greater endorsement of leadership/authority and exhibitionism/entitlement.

To measure individual differences in one’s drive for muscularity we used the 5-item Drive for Muscularity Scale [31]. Participants reported their endorsement (1 = *never*; 6 = *always*) of items like “I think I would feel more confident if I had more muscle mass/body tone”. Items were averaged to create an index of drive for muscularity, with higher scores indicating a greater drive for muscularity. To measure individual differences in one’s drive for thinness we used the 17-item Drive for Thinness Scale [32]. Participants reported their endorsement (1 = *never*; 6 = *always*) of items like “I am preoccupied with the desire to be thinner”. Items were averaged to an create index of drive for thinness, with higher scores indicating a greater drive for thinness.

To measure the amount of eating disorder symptoms participants experienced, we used the 12-item Eating Disorder Examination Questionnaire–Short [33]. Participants indicated their frequency of symptoms per week (0 = *0 days*; 3 = *6–7 days*) with items like “have you had a definite fear that you might gain weight?”. Items were averaged to create an index of eating disorder pathology with higher scores indicating more eating disorder symptoms.

To measure individual differences in current and desired bodies we used the Sex-Specific Somatomorphic Matrixes for men and women [34, 35]. In both cases, men and women were shown a bi-dimensional pictorial figural rating scale depicting a grid of 34 same-sex figures, ranging from underweight-to-obese, and underweight-to-hypermuscular. Participants selected their (sex-specific) current and desired bodies, scored for body fat and muscularity (1 = *an underweight body/body with little muscularity*; 10 = *an obese/hyper-muscular body*). Selections were averaged to create an index of current and desired bodies with higher scores indicating more current/desired body fat and muscularity.

## Results

In Table 1 we report the correlations within and between factors of narcissism and measures of body image concerns, and sex differences in both. All narcissistic factors (i.e., agentic extraversion, antagonism, narcissistic neuroticism, leadership/authority, and exhibitionism/entitlement) were associated with greater drive for thinness (except for leadership/authority) and drive for muscularity, more eating disorder symptoms, greater desired body fat (except for leadership/authority), and greater current muscularity. Current body fat was negatively correlated with agentic extraversion and exhibitionism/entitlement, and a greater desired muscularity was associated with more antagonism. Men were better characterized by antagonism and had a stronger drive for muscularity, and a higher degree of desired muscularity than women, whereas women had a stronger drive for thinness than men.

**Table 1:** Descriptive statistics, correlations within and between factors of narcissism and measures of body image, and sex differences for both.

	1	2	3	4	5	6	7	8	9	10	11	12	13
1. Antagonism													
2. Agentic extraversion	.68**												
3. Leadership/Authority	32**	.61**											
4. Exhibitionism/Entitlement	.56**	.66**	.62**										
5. Narcissistic neuroticism	.76**	.49**	.16**	.40**									
6. Drive for thinness	.52**	.25**	.02	.20**	.59**								
7. Drive for muscularity	.73**	.59**	.22**	.49**	.69**	.58**							
8. Eating disorder symptoms	.69**	.52**	.16**	.43**	.67**	.70**	.74**						
9. Current body fat	-.09	-.17**	-.09	-.17**	.01	.24**	-.06	.08					
10. Desired body fat	.20**	.10*	.06	.17**	.18**	.25**	.16**	.28**	.50**				
11. Current muscularity	.18**	.23**	.16**	.16**	.14**	.15**	.18**	.15**	-.17**	.04			
12. Desired muscularity	.11*	.04	.05	.01	.09	.07	.11*	-.02	.06	-.08	.46**		
13. Body mass index	-.05	-.09	.02	-.06	-.01	.19**	-.02	.10*	.39**	.23**	.07	.12*	
Cronbach’s α	.93	.94	.72	.74	.87	.82	.95	.93	-	-	-	-	-
Overall (*N* = 430): *M* (SD)	3.46 (1.33)	4.07 (1.12)	6.07 (3.01)	5.59 (3.32)	3.27 (0.83)	3.32 (0.83)	3.37 (1.27)	1.19 (0.77)	5.35 (1.95)	4.53 (1.80)	5.33 (2.23)	5.94 (2.25)	26.57 (7.33)
Men (*n* = 275): *M* (SD)	3.58 (1.30)	4.14 (1.08)	6.27 (2.82)	5.80 (3.20)	3.29 (0.83)	3.24 (0.81)	3.46 (1.21)	1.18 (0.78)	5.41 (1.95)	4.66 (1.88)	5.26 (2.17)	6.16 (2.13)	26.28 (6.66)
Women (*n* = 155): *M* (SD)	3.27 (1.38)	3.97 (1.17)	5.71 (3.29)	5.21 (3.50)	3.23 (0.83)	3.46 (0.85)	3.19 (1.35)	1.19 (0.75)	5.24 (1.95)	4.30 (1.65)	5.46 (2.34)	5.54 (2.41)	27.05 (8.34)
*t*-test	2.34*	1.50	1.86	1.80	0.70	-2.65**	2.15*	0.92	0.86	1.95_a_	-0.91_a_	2.77**_a_	-1.03_a_
Cohen’s *d*	0.23	0.15	0.19	0.18	0.07	-0.26	0.21	0.01	0.09	0.20	-0.09	0.28	-0.10

*Note*.

_a_ Current and desired muscularity and body fat do not strictly compare the same things in the sexes so the interpretation of the corresponding effects should be made with caution; for Body mass index: Men (*n* = 265), Women (*n* = 154) caused by missing data.

* *p* < .05

** *p* < .01

Given that all narcissistic aspects of personality were correlated (see Table 1), we ran seven standard multiple regressions to control for the shared variance (i.e., one for each of the measures of body image. The reported correlations are based on residualized values, indicating the influence of three factors of narcissism (namely, antagonism, agentic extraversion, narcissistic neuroticism) on drive for thinness, accounting for a substantial amount of variance (38%; *F* = 87.27, *p* < .01). The findings demonstrate that antagonism and narcissistic neuroticism exhibit positive associations with drive for thinness, while agentic extraversion shows a negative association. Similarly, when considering five factors of narcissism (antagonism, agentic extraversion, narcissistic neuroticism, leadership/authority, exhibitionism/entitlement), the residualized correlations underscore their impact on drive for muscularity (61%; *F* = 131.16, *p* < .01) and eating disorder symptoms (54%; *F* = 100.29, *p* < .01). Specifically, leadership/authority displays a negative correlation with drive for muscularity and eating disorder symptoms, while the remaining factors exhibit positive correlations with both. In contrast, from narcissistic factors: exhibitionism/entitlement accounted for (relatively) trivial variance in current body fat (3%; *F* = 13.35, *p* < .01) being negatively related to it; antagonism accounted for (relatively) trivial variance in desired body fat (4%; *F* = 17.61, *p* < .01) being positively related to it; agentic extraversion accounted for (relatively) trivial variance in current muscularity (5%; *F* = 22.91, *p* < .01) being positively related to it; antagonism accounted for (relatively) trivial variance in desired muscularity (1%; *F* = 4.83, *p* < .05) being positively related to it. Notably, none of narcissistic factors predicted body mass index.

In Table 2 we compared the correlations in men and women. In all factors of narcissism, except for leadership/authority, drive for thinness was more strongly correlated in men than in women. Moreover, antagonism was associated with current body fat more negatively in women than in men, but it was more positively associated with desired body fat in men characterized by more antagonism, and exhibitionism/entitlement. Interestingly, leadership/authority was more positively associated with eating disorder symptoms among women. These results, which are in alignment with previous research findings (Li et al., 2010) [38], highlight the importance of understanding the role of intrasexual competition in the development of eating disorders among women. That said, the correlations were, on average, similar suggesting no wide-spread moderation (*M*_z_ = 0.79, *SD* = 1.52; *Range* = -2.38 to 4.91).

**Table 2:** Correlations between measures of narcissism and measures of body image in both men (*n* = 275) and women (*n* = 155).

	Antagonism		Agentic		Leadership/		Exhibitionism/		Narcissistic	
			extraversion		Authority		Entitlement		neuroticism	
	M/W	*z*	M/W	*z*	M/W	*z*	M/W	*z*	M/W	*z*
Drive for thinness	.68**/.33**	4.91**	.38**/.06	3.36**	.06/-.02	0.79	.32**/.05	2.78**	.67**/.49**	2.89**
Drive for muscularity	.77**/.68**	1.89	.62**/.54**	1.04	.17**/.28**	-1.15	.46**/.53**	-0.92	.68**/.70**	-0.38
Eating disorder symptoms	.72**/.64**	1.48	.53**/.50**	0.40	.07/.30**	-2.36*	.43**/.43**	<0.01	.67**/.66**	0.18
Current body fat	.02/-.29**	3.15**	-.12*/-.26**	1.44	-.05/-.14	0.90	-.11/-.29**	1.86	.10/-.16	2.58**
Desired body fat	.28**/.03	2.54*	.14*/.01	1.29	.07/.03	0.40	.24**/.03	2.12*	.21**/.12	0.91
Current muscularity	.25**/.10	1.53	.26**/.18*	0.83	.15*/.20*	-0.51	.17**/.16*	0.10	.16*/.11	0.50
Desired muscularity	.10/.08	0.20	.03/.04	-0.10	-.01/.09	-0.99	-.11/.13	-2.38*	.10/.07	0.30
Body mass index	-.01/-.09	0.79	-.11/-.06	-0.50	.03/.01	0.20	-.09/-.02	-0.69	-.01/-.02	0.10

*Note*. *z* is Fisher’s *z* that was calculated online (http://quantpsy.org/corrtest/corrtest.htm), it compares independent correlations.

* *p* < .05

** *p* < .01

Given that all factors of narcissism were correlated (see Table 1), we compared the relative magnitudes of their correlations with the body image variables while controlling for their shared variances with Steiger’s *z* tests, revealing several effects (Table 3). Compared to all three factors—agentic extraversion, leadership/authority, and exhibitionism/entitlement—antagonism correlated positively and more strongly with drive for thinness, drive for muscularity, and eating disorder symptoms. Additionally, compared to agentic extraversion, antagonism correlated positively and more strongly with current body fat, and desired body fat, but weaker with current muscularity; compared to leadership/authority, antagonism correlated positively and more strongly with desired body fat; compared to exhibitionism/entitlement, antagonism correlated positively and more strongly with desired muscularity. On the other hand, compared to narcissistic neuroticism, antagonism was more negatively correlated with drive for thinness and current body fat. Compared to leadership/authority, and exhibitionism/entitlement, agentic extraversion correlated positively and more strongly with drive for thinness (except for comparison with exhibitionism/entitlement), drive for muscularity, and eating disorder symptoms. Additionally, compared to leadership/authority, agentic extraversion correlated negatively and more strongly with body mass index. Lastly, compared to agentic extraversion, leadership/authority, and exhibitionism/entitlement, narcissistic neuroticism correlated positively and more strongly with drive for thinness, drive for muscularity, eating disorder symptoms, and current body fat (for the last one- except for comparison with leadership/authority).

**Table 3:** Steiger’s *z* tests comparing the correlations between measures of narcissism with measures of body image.

	ANT→EX	ANT→LA	ANT→EE	ANT→NN	EX→LA	EX→EE	EX→NN	LA→NN	EE→NN
Drive for thinness	7.86**	9.83**	7.91**	-2.59**	5.50**	1.29	-8.18**	-10.45**	-8.60**
Drive for muscularity	5.23**	11.85**	7.42**	1.81	10.11**	3.10**	-2.95**	-9.53**	-5.20**
Eating disorder symptoms	5.91**	11.74**	7.55**	0.43	9.41**	2.64**	-4.48**	-10.04**	-5.96**
Current body fat	2.09*	<0.01	1.78	-2.99**	-1.89	<0.01	-3.72**	-1.60	-3.43**
Desired body fat	2.62**	2.52*	0.68	0.61	0.94	-1.78	-1.66	-1.94	-0.19
Current muscularity	-2.13*	0.36	0.45	1.21	1.68	1.80	1.89	0.32	0.38
Desired muscularity	1.82	1.07	2.43*	0.60	-0.23	1.00	-1.03	-0.64	-1.70
Body mass index	1.04	-1.24	0.22	-1.19	-2.58**	-0.75	-1.64	0.48	-0.94

*Note*. Steiger’s *z* was calculated online (http://quantpsy.org/corrtest/corrtest2.htm), it compares dependent correlations. ANT = Antagonism, LA = Leadership/Authority, EE = Exhibitionism/Entitlement, EX = Assertive extraversion, NN = Narcissistic neuroticism

* *p* < .05

** *p* < .01

## Discussion

Our study provided an extensive investigation of narcissism and body image, and, to our knowledge, the first collective examination of different aspects of narcissism postulated by Trifurcated Model of Narcissism (and more) with eating disorder symptoms and current and desired body image, and the first comparison of these associations in men and women. Generally, all narcissistic traits (i.e., agentic extraversion, antagonism, narcissistic neuroticism, leadership/authority, and exhibitionism/entitlement) were positively associated with greater drive for thinness (except for leadership/authority) and drive for muscularity, more eating disorder symptoms, greater desired body fat (except for leadership/authority), and greater current muscularity, suggesting that narcissistic individuals (regardless of their dominant trait) consider their physical appearance to be important, and therefore likely hold attitudes and engage in behaviors that reflect an aspiration toward obtaining the ideal body.

More specifically, when controlling for the shared variance in narcissism, we found that agentic extraversion was negatively correlated with drive for thinness, and that leadership/authority was negatively correlated with drive for muscularity and eating disorder symptoms, suggesting that eating disorder psychopathology may serve these narcissistic factors differently. While agentic extraversion and especially leadership/authority aspect of narcissism may be seen as protective factor for eating disorders, the opposite effect may be observed for narcissistic neuroticism. The links between narcissistic neuroticism (i.e., vulnerable narcissism) and eating disorder psychopathology evidenced in our study and others [36] likely reflect a fear of negative evaluations and, therefore, attempts to avoid or minimize disapproval through obtaining the ideal thin and muscularly toned body (Hendin & Cheek, 1997). Attitudes and behaviors that drive one to obtain the ideal body would also accommodate a sense of superiority and the opportunity to devalue or look down upon individuals with less-than-ideal bodies, thus serving antagonism [4]. On the other hand, agentic extraversion–assertive and self-enhancing behaviors aimed to generate more social admiration and to boost one’s ego [4]–could also be fulfilled by obtaining the ideal body.

Regarding sex differences, our results, extend previous work suggesting that in women, narcissistic admiration (i.e., agentic extraversion) and rivalry (i.e., antagonism) can predict drive for thinness and drive for muscularity [19]. We found that antagonism was associated with current body fat more negatively in women, but it was more positively associated with desired body fat in (characterized by antagonism and exhibitionism/entitlement) men. Moreover, in all factors of narcissism (except for leadership/authority) drive for thinness was more strongly correlated in men than in women. Interestingly, leadership/authority was more positively associated with eating disorder symptoms among women. These findings may suggest that narcissistic men (except for those characterized by leadership/authority) are driven to thinness more than narcissistic women and that they will probably be more likely to display behavior aimed at staying thin than average person. Conversely, our findings indicate that women exhibiting traits of leadership/authority may experience a greater prevalence of eating disorder symptoms. This underscores the potential significance of intrasexual competition in the context of eating disorder development among women.

There may be several ways to understand such sex differences. First, sex differences may be under sociocultural influence. It is well-documented that women, in particular, face societal pressures to conform to thinness ideals, which could amplify the associations between certain facets of narcissism and drive for thinness [32]. Second, if narcissism is a trait that facilitates reproductive success and intrasexual competition [37], body image concerns may be part of an internal monitoring system around one’s mate value. That is, by maximizing the appeal of their physical form, narcissistic men and women may better find romantic and sexual partners. For instance, our finding that leadership/authority was more positively associated with eating disorder symptoms among women suggests that specific facets of narcissism may have varying impacts on different sexes. This may be manifestation of intrasexual competition among women [38]. Third, alternatively, both narcissism and body image concerns may be manifestations of psychopathology that manifest differently in both sexes. That is, while male and female brains may break down for similar reasons, the proverbial devil is in the details; the manner by which narcissism and body image disturbances are expressed may differ in men and women. This suggests that men and women may exhibit different patterns of body image concerns based on their narcissistic traits. For example, in our study, antagonism was negatively associated with current body fat in women, while it was positively associated with desired body fat in men. Future studies should more fully explore these sex-differentiated effects and attempt to disentangle not just the patterns but also the various influences upon them like cultural conditioning and evolved mechanisms. Comparing across the sexes, men reported greater antagonism than women. Men also reported greater drive for muscularity than women, but less drive for thinness. Somewhat surprisingly, men’s drive for thinness was more strongly correlated with all narcissistic factors (apart from leadership/authority). Given that generally, women in our sample had a higher drive for thinness than men, this result might reflect a ceiling effect (i.e., a weaker association between drive for thinness and narcissism in women because of a higher average drive for thinness). Whilst as drive for thinness is less pronounced in men, it may be only narcissistic men who put more emphasis into lowering body fat. Alternatively, this difference may reflect differences in sexual selection. For instance, to receive attention and validation, and to be attractive, narcissistic men may place greater emphasis on the importance of obtaining and maintaining a lean, desirable body [39].

When comparing the strength of associations, the top three most important aspects of narcissism for body image were antagonism, narcissistic neuroticism, and agentic extraversion, suggesting that they may be the most important predictors (within narcissism) of body image concerns. These results, in part, correspond to prior studies that, for instance, revealed a link between vulnerable narcissism (i.e., narcissistic neuroticism) and thinness concerns [15, 19, 40], drive for muscularity [19], and eating disorder symptoms [36, 40, 41] in women. Antagonism, often regarded as a central component of narcissism along with exhibitionism and entitlement, has been previously linked to higher levels of impulsivity, anger, pathological vulnerability, and negative self-evaluations [4]. Therefore, our findings align with the anticipated outcomes associated with antagonism, which is considered a core element in various psychopathological conditions [1]. With regards to agentic extraversion, including leadership and authority, we anticipated it would be the most adaptive narcissistic factor in relation to one’s body image concerns. Our findings could be interpreted as providing partial support for this perspective.

When examining choices for both current and desired body types, some trends were identified. For instance, current muscularity was weakly positively correlated with all aspects of narcissism, reflecting that narcissistic people see themselves as closer to the muscular ideal than those who are less narcissistic. Narcissists higher on agentic extraversion and on exhibitionism/entitlement saw themselves as less fat. Apart from leadership/authority, all kind of narcissistic individuals reported greater desired body fat. Nevertheless, only antagonism exhibited a correlation with a stronger desire for muscularity. Perceptions of one’s body as fat or less than ideal may reflect the feelings of inadequacy, insecurity, and low self-esteem that especially characterize vulnerable narcissists [42]. Furthermore, perception, by fitting with a more ideal body shape, may help to protect the ego from external threat, and allow for downward comparisons to the bodies of others that are perceived as fatter [4]. However, correlations across all current and desired body selections were modest. This might in part be the result of a lack of common-method variance [43] across our measures of narcissism and current/desired body selections (i.e., Likert-style questions vs. pictorial stimuli selection), thus weakening our current/desired body selection correlations relative to those observed using Likert-style measures.

In our study, we observed that different aspects of narcissistic traits were correlated with body image concerns in distinct ways. This implies that not all facets of narcissism have uniformly maladaptive associations with body image concerns. Some of these aspects may exhibit more adaptive qualities, suggesting that the impact of narcissistic traits on body image outcomes should be critically appraised. For example, our findings indicated that agentic extraversion, along with leadership/authority, appeared to play a more adaptive role compared to other facets of narcissism in relation to body image concerns. This suggests that individuals higher in these aspects may demonstrate more positive body image perceptions or attitudes. It is important to note that this does not imply that all aspects of narcissism are inherently adaptive, but rather highlights the potential for variability in their associations with body image concerns. The distinction between adaptive and maladaptive aspects of narcissism holds clinical and practical significance. Understanding the specific aspects of narcissism that may have adaptive qualities can inform intervention strategies and treatment approaches. For instance, interventions targeting individuals characterized by agentic extraversion and leadership/authority may benefit from promoting body positivity and healthy body image attitudes. Recognizing the potential for adaptive qualities within narcissistic traits can help tailor interventions to individuals’ unique characteristics and needs. On the other hand, it is crucial to remain cautious when interpreting the findings and avoid generalizations. Although certain sub-aspects of narcissism may exhibit more adaptive associations with body image concerns, it is essential to consider the overall context and the potential aversive consequences of narcissistic traits. The clinical/practical implications involve striking a balance between acknowledging the potential adaptive qualities and addressing the maladaptive aspects of narcissism in body image-related interventions.

### Limitations & conclusions

In this study we engaged in a broadband assessment of how narcissism, in several manifestations, may relate to individual differences in body image, also in several manifestations. Despite our approach, our study was, nevertheless, limited in its reliance on Americans [44], and the use of only online methods for data collection [45]. Our data was also potentially heteronormative, failing to take into consideration differences in body image concern in sexual minorities from either sex [46]. Also, online convenience sampling, while easy and cost-effective, has limitations in terms of generalizability to the broader population. Importantly, the data for this study was collected in 2021. The COVID-19 (i.e., Coronavirus Disease 2019) pandemic at that time introduced unique and widespread stressors, including social isolation, changes in daily routines, and increased reliance on virtual interactions. These factors may have influenced individuals’ perceptions of body image and levels of narcissism. For example, extended periods of isolation and increased screen time might have affected body image self-perception because of altered social comparison dynamics. Likewise, the heightened stress and uncertainty associated with the pandemic could influence narcissistic tendencies. By acknowledging these contextual factors in our study, we can better interpret our findings and understand their relevance in the context of the potential correlation between the pandemic and psychological well-being. Another methodological constraint in our study pertains to the potential for false positives or the amplification of significant findings due to numerous pairwise comparisons, warranting careful interpretation. Additionally, dedicated tools have already been developed to evaluate various aspects of narcissism simultaneously (e.g., Five-Factor Narcissism Inventory Short Form [47]) which could capture narcissistic factors in even greater detail. A further potential limitation is our lack of consideration of self-esteem, which shares a link with both body dissatisfaction [48] and narcissism [49]. And last, several other subclinical personality pathologies might be relevant in study of body image concerns, like obsessive compulsive disorder/personality [50] and borderline personality disorder [51].

Despite these limitations, we have provided the most robust account (to date) of how several manifestations of both narcissism and body image disturbances are related. Notably, individual differences in different aspects of narcissism appear to be important in understanding different manifestations of body image concerns, as well as other eating disorder-related behaviors. Generally, we found that narcissistic traits may be risk factors for developing and perpetuating body image concerns, and therefore should be a considered in assessment, formulation, diagnosis, and treatment of eating disorders.
